# Rate of various access sites for temporary transvenous pacing and different outcomes at Lady Reading Hospital, Peshawar Pakistan

**DOI:** 10.12669/pjms.39.4.7467

**Published:** 2023

**Authors:** Muhammad Adil, Sher Bahadar Khan, Muhammad Shahbaz Khan, Zair Hassan

**Affiliations:** 1 Muhammad Adil, MBBS, FCPS Cardiology Assistant Professor Department of Cardiology, Medical Teaching Institute, Lady Reading Hospital, Peshawar, KPK, Pakistan; 2 Sher Bahadar Khan, MBBS, FCPS Cardiology, Fellowship Intervention Cardiology (IJN) Associate Professor Department of Cardiology, Medical Teaching Institute, Lady Reading Hospital, Peshawar, KPK, Pakistan; 3 Muhammad Shahbaz Khan, MBBS Postgraduate Resident Department of Cardiology, Medical Teaching Institute, Lady Reading Hospital, Peshawar, KPK, Pakistan; 4 Zair Hassan, MBBS Postgraduate Resident Department of Cardiology, Medical Teaching Institute, Lady Reading Hospital, Peshawar, KPK, Pakistan

**Keywords:** Temporary pacemaker, Temporary pacemaker complications, Complete heart block, Sinus node disease

## Abstract

**Objective:**

To evaluate the various temporary transvenous pacemaker (TPM) access sites, its indications, procedural complications, and outcomes of patients.

**Methods:**

This prospective study conducted in a tertiary care hospital of Peshawar, included 100 patients, who underwent TPM for any reasons, via the trans jugular, subclavian, or trans-femoral route. The duration of the study was from October 1^st^, 2021 to March 31^st^, 2022. The demographic, procedure -related complications, causes of complete heart block and in hospital outcomes were recorded.

**Results:**

Of the 100 patients who underwent temporary transvenous pacing, 56%were males and 44% were females, with an age range of 46-80 years. In majority of the patients, (N =54) internal jugular vein was used as the venous access site followed by the subclavian vein. (N=24). Coronary artery disease was prevalent in 42% of the patients. 50% had complete AV block, 19% had symptomatic second-degree block, and 10% had sinus nodal diseases. Seventy three percent of the patients needed TPM implantation on an emergency basis, which is statistically significant (p=0.009). Almost 40% of the patient ultimately underwent a permanent pacemaker. Out of 100 patients, 16 patients expired. The major procedure related complications were bleeding 16% overall at the puncture site and 14.8% in the internal jugular group. Other complications were local infection 13% at the insertion site followed by hemopericardium 3%, in the internal jugular group.

**Conclusion:**

Atrioventricular block is the commonest indication for temporary pacing in our study. The average time the TPM remained in place was significantly higher in the trans jugular approach group along with a higher complication rate in this group.

## INTRODUCTION

Temporary Transvenous Pacemaker (TPM) is a lifesaving procedure in patients with symptomatic atrioventricular (AV) blocks and serves as a bridge to permanent pacemaker (PPM) implantation. TPM is indicated for various symptoms caused by third-degree AV block, bradyarrhythmia, and life-threatening tachyarrhythmias.^1^-^4^ Temporary pacemakers (TPM) are almost always performed in acute emergencies. One of its most common indications is the complete heart block (3rd) degree or the sinus node dysfunction with hemodynamic instability.

TPM can be implanted using different venous routes. All the main venous access sites have been accessed at different times for the purpose of temporary pacing. However, every venous access site is associated with its own complications, which include local discomfort, pneumothorax, instability of the implanted lead, and infection.^5^ The right internal jugular vein is the most favored intravenous access site for unguided temporary transvenous pacing.^6^ The complication rate can differ according to the venous access site but alternatively there is some evidence that suggests that the complication rate during the procedure depends more on the expertise of the operator rather than the anatomical venous access site as suggested by a retrospective study conducted in Aga Khan University.^3^ In another prospective study about temporary pacemaker placement through the subclavian vein demonstrated that the supraclavicular approach as compared to the more established infra-clavicular was comparable in terms of accessibility and in complication rate but had the advantage of requiring lesser time and hence more chances of success.^7^ The temporary pacemaker serve as a critical interim therapy and delay between transition in the temporary pacemaker itself and in upgradation to the permanent pacemaker therapy can have serious complications and adverse outcomes for the patients including syncope and asystole as highlighted by Irfan and his colleagues in their study.^8^

Our study evaluated the various indication, different access sites, complications and outcomes of the TPM placement which were performed by post graduate trainees of the cardiology department in Peshawar Pakistan. Temporary venous pacing can be performed from the various venous access point; all these different sites, are associated with its own potentially serious complications and different adverse outcomes; such a study comparing the outcomes from the venous access sites has not been done before in our country and the lack of local data was the main rationale in conducting our study.

## METHODS

A cross-sectional study that was conducted in the Cardiology Department, Lady reading hospital, MTI Peshawar KPK after taking the ethical approval from the ethics committee reference no. 345/LRH/MTI dated 26/09/2021. Informed consent was taken either from patients or their relatives (in case patients were not oriented due to complete heart block). The duration of the study was from 1^st^ October,2021 to 31^st^, March 2022, and included all those patients with symptomatic heart block (either complete heart block or 2^nd^ degree heart block) due to any cause via nonprobability consecutive sampling technique. A total of 100 patients were considered for the final analysis after fulfilling the inclusion criteria.

The patients having congenital heart blocks were excluded from the study. The total time taken for the completion of the procedure was time from the first venous puncture to the successful ventricular pacing. All the temporary pacemakers were inserted under fluoroscope guidance. The confirmation for successful ventricular pacing was performed via 12 lead EKG after the procedure as well as using the cardiac monitor during the procedure,

Data were entered and analyzed using SPSS V 23. Frequency and percentages were used for categorical data. Pearson’s chi-squared test was used to determine the association between two variables. For all tests, the cut-off for statistical significance was set at p-value 0.05.

## RESULTS

The majority of the patients were male 56(56.00%). Nearly half of the patients were in the age group of 70-80 years. The study patients were divided into three groups based on the TPM procedure access type. The majority were accessed through the internal jugular vein. The proportion of demographics such as age, gender, anthropometric, and clinical characteristics that is body mass index (BMI), smoking, and drug history were not significantly different between the study groups (TPM procedure access type). Hypertension, diabetes, coronary artery disease, and dyslipidemia, were more common in the internal jugular group than in the femoral and subclavian groups however no statistically significant association was observed as shown in Table-I.

**Table-I:** The background characteristics of the study participants across TPM access type.

		Total N (%)	TPM procedure access type.						*p*-value

			Subclavian N=24		Internal jugular N=54		Femoral n=22		

			N	%	N	%	N	%	
Age	<50	5(5.00)	2	8.33	3	5.56	0	0.00	0.541
	50-60	9(9.00)	1	4.17	6	11.11	2	9.09	
	60-70	31(31)	10	41.67	13	24.07	8	36.36	
	70-80	55(55)	11	45.83	32	59.26	12	54.55	
Gender	Male	56(56)	11 13	45.83 54.17	32 22	59.26 40.74	13 9	59.09 40.91	.516
	Female	44(44)							
BMI	Obese	28(28)	7	29.17	15	27.78	6	27.27	0.988
	Normal	72(72)	17	70.83	39	72.22	16	72.73	
Smoking	Yes	14(14)	5	20.83	6	11.11	3	13.64	0.520
	No	86(86)	19	79.17	48	88.89	19	86.36	
Drugs	Yes	16(16)	4	16.67	10	18.52	2	9.09	0.593
	No	84(84)	20	83.33	44	81.48	20	90.91	
Chronic pulmonary disease	Yes	6(6)	1	4.17	4	7.41	1	4.55	0.813
	No	94(94)	23	95.83	50	92.59	21	95.45	
Chronic renal failure	Yes	27(27)	7	29.17	14	25.93	6	27.27	0.956
	No	73(73)	17	70.83	40	74.07	16	72.73	
Congestive heart failure	Yes	14(14)	4	16.67	10	18.52	0	0.00	0.098
	No	86(86)	20	83.33	44	81.48	22	100.00	
Coronary artery disease	Yes	42(42)	9	37.50	22	40.74	11	50.00	0.666
	No	58(58)	15	62.50	32	59.26	11	50.00	
Diabetes mellitus	Yes	57(57)	16	66.67	29	53.70	12	54.55	0.546
	No	43(43)	8	33.33	25	46.30	10	45.45	
Dyslipidemia	Yes	23(23)	6	25.00	16	29.63	1	4.55	0.060
	No	77(77)	18	75.00	38	70.37	21	95.45	
Hypertension	Yes	65(65)	18	75.00	32	59.26	15	68.18	0.380
	No	35(35)	6	25.00	22	40.74	7	31.82	
History of myocardial infarction	Yes	19(19)	4	16.67	12	22.22	3	13.64	0.650
	No	81(81)	20	83.33	42	77.78	19	86.36	
Previous PCI	Yes	11(11)	4	16.67	6	11.11	1	4.55	0.422
	No	89(89)	20	83.33	48	88.89	21	95.45	
Previous CABG	Yes	3(3)	1	4.17	2	3.70	0	0.00	0.643
	No	97(97)	23	95.83	52	96.30	22	100.00	

Complete heart block 50 (50%) was the most common indication for TPM placement among the groups followed by second -degree heart block and sinus node disease, but the emergency indication profile was significantly different between the TPM procedure access type (P=0.009). Complete heart block was more common in the internal jugular approach group than the others two groups, whereas background acute myocardial infarction was more common in the internal jugular groups than the subclavian and transfemoral approaches. The procedure time for TPM placement was shorter in the internal jugular group compared to the other two groups.

The median duration of temporary pacing was less than five days in all three groups, whereas 5-10 days duration was almost similar in both the subclavian and internal jugular groups and more than the femoral group. The survival rate was significantly more on the internal jugular approach compared with the other two groups of the total, 13 patients underwent reposition of the TPM lead wire during temporary pacing in the internal jugular 13(24.07%), subclavian 11(45.83%), and femoral groups 9(40.90%), respectively (Table-III). The most common cause of repositioning the wire was capturing failure in all three groups. Among the procedure -related complications, the puncture site bleeding and hemopericardium were higher in the internal jugular group compared with the other groups.

**Table-II:** Indication, Procedure Related Details and Outcome According to TPM Procedure Access Type.

		Total N(%)	TPM procedure access type.						*p*-value

			Subclavian		Internal jugular		Femoral		

			N	%	N	%	N	%	
Indication for TPM	Second degree AV block	19(19)	7	29.17	10	18.52	2	9.09	0.181
	Complete heart block	50(50)	8	33.33	29	53.70	13	59.09	
	Sinusnode disease	10(10)	4	16.67	2	3.70	4	18.18	
	Symptomatic bradycardia	11(11)	3	12.50	7	12.96	1	4.55	
	Bradycardia related to MI	8(8)	1	4.17	6	11.11	1	4.55	
	During cardiac surgery or PCI	2(2)	1	4.17	0	0.00	1	4.55	
Emergency indication of TPM	Yes	73(73)	13	54.17	46	85.19	14	63.64	0.009*
	No	27(27)	11	45.83	8	14.81	8	36.36	
Background of acute MI	Yes	39(39)	7	29.17	26	48.15	6	27.27	0.126
	No	61(61)	17	70.83	28	51.85	16	72.73	
Ease of access	First pass	64(64)	17	70.83	35	64.81	12	54.55	0.838
	Multiple attempts at one site	32(32)	6	25.00	17	31.48	9	40.91	
	Multiple attempts >1 site	4(4)	1	4.17	2	3.70	1	4.55	
Need for permanent pace maker	Yes	40(40)	11	45.83	18	33.33	11	50.00	0.324
	No	60(60)	13	54.17	36	66.67	11	50.00	
Duration of TPM	< 5 days	76(76)	16	66.67	43	79.63	17	77.27	0.224
	5-10days	21(21)	8	33.33	8	14.81	5	22.73	
	>10 days	3(3)	0	0.00	3	5.56	0	0.00	
Procedure time (minutes)	<5	9(9)	3	12.50	6	11.11	0	0.00	0.404
	5-10	46(46)	9	37.50	24	44.44	13	59.09	
	10-15	45(45)	12	50.00	24	44.44	9	40.91	
Patient outcome at the end of TPM	Survive	84(84)	21	87.50	42	77.78	21	95.45	0.141
	Expired	16(16)	3	12.50	12	22.22	1	4.55	

The correlation here is done between the patient outcome at the end of the TPM procedure and the venous access site used for the procedure which doesn't have a significant correlation. not between the patients who survived and those who didn't survive after TPM.

**Table-III:** Complication related to procedure and cross tabulation according to TPM procedure access type.

		Total N (%)	TPM procedure access type.						*p*-value

			Subclavian		Internal jugular		Femoral		

			N	%	N	%	N	%	
Arterial puncture	Yes	16(16)	4	16.67	8	14.81	4	18.18	0.931
	No	84(84)	20	83.33	46	85.19	18	81.82	
Pneumothorax	Yes	1(1.0)	1	4.17	0	0.00	0	0.00	0.202
	No	99(99)	23	95.83	54	100.00	22	100.00	
Hemopericardium	Yes	3(3)	0	0.00	3	5.56	0	0.00	0.268
	No	97(97)	24	100.00	51	94.44	22	100.00	
Repositioning	Yes	33(33)	11	45.83	13	24.07	9	40.91	0.113
	No	67(67)	13	54.17	41	75.93	13	59.09	
Local infection	Yes	13(13)	4	16.67	3	5.56	6	27.27	0.032*
	No	87(87)	20	83.33	51	94.44	16	72.73	
> than one complication	Yes	1(1)	1	4.17	0	0.00	0	0.00	0.202
	No	99(99)	23	95.83	54	100.00	22	100.00	

**Table-IV:** Characteristics of Survivors and Non Survivors.

		Outcome				*P-*value

		Survive N=84		Expired N=16		

		N	%	N	%	
Age	<50	4	4.76	1	6.25	0.392
	50-60	6	7.14	3	18.75	
	60-70	28	33.33	3	18.75	
	70-80	46	54.76	9	56.25	
Gender	Male	46	54.76	10	62.50	0.568
Indication for TPM	Second degree AV Block	13	15.48	6	37.50	0.175
	CHB	43	51.19	7	43.75	
	Sinusnode disease	10	11.90	0	0.00	
	Symptomatic bradycardia	10	11.90	1	6.25	
	bradycardia related to MI	7	8.33	1	6.25	
	During cardiac surgery or other procedure	1	1.19	1	6.25	
Emergency indication of TPM	Yes	61	72.62	12	75.00	0.844
Background of acute MI	Yes	30	35.71	9	56.25	0.123
Site of access for TPM	Subclavian	21	25.00	3	18.75	0.141
	Internal jugular vein	42	50.00	12	75.00	
	Femora vein	21	25.00	1	6.25	
Ease of access	First pass	51	60.71	13	81.25	0.260
	multiple attempts at one site	29	34.52	3	18.75	
	multiple attempts >1 site	4	4.76	0	0.00	
Need for ppm	Yes	35	41.67	5	31.25	0.436
Duration of TPM	less than 5 days	68	80.95	8	50.00	0.007*
	5-10days	15	17.86	6	37.50	
	more than 15 days	1	1.19	2	12.50	
Procedure time (minutes)	1-5	4	4.76	5	31.25	0.002*
	5-10	39	46.43	7	43.75	
	10-15	41	48.81	4	25.00	
Arterial puncture	Yes	13	15.48	3	18.75	0.743
Pneumothorax	Yes	0	0.00	1	6.25	0.021*
Hemopericardium	Yes	1	1.19	2	12.50	0.015*
Repositioning	Yes	31	36.90	2	12.50	0.057
Local infection	Yes	13	15.48	0	0.00	0.092
>One complication	Yes	1	1.19	0	0.00	0.661
Chronic pulmonary disease	Yes	6	7.14	0	0.00	0.270
Chronic renal failure	Yes	19	22.62	8	50.00	0.024*
Congestive heart failure	Yes	10	11.90	4	25.00	0.166
Coronary artery disease	Yes	30	35.71	12	75.00	0.004*
Diabetes mellitus	Yes	46	54.76	11	68.75	0.300
Dyslipidemia	Yes	17	20.24	6	37.50	0.133
Hypertension	Yes	54	64.29	11	68.75	0.731
Obesity	Obese	22	26.19	6	37.50	0.356
Previous MI	Yes	14	16.67	5	31.25	0.173
Previous PCI	Yes	6	7.14	5	31.25	0.005*
Previous CABG	Yes	2	2.38	1	6.25	0.406
Smoking	Yes	9	10.71	5	31.25	0.030*
Drugs	Yes	14	16.67	2	12.50	0.677

Local infection was significantly higher in the femoral group compared to others non-procedure - related complications such as lead repositioning were higher in the internal group. Those with the age 70-80 years were the highest among non-survivors. Similarly, the majority of the patients with complete heart block and those presented for emergency indications of TPM did not survive. On the other hand, those with a duration of TPM, 80.95% had a higher survival rate and was significantly differed in survivors and non-survivors. Those with, pneumothorax, hemopericardium, chronic renal failure, and coronary artery diseases had a high mortality rate. Moreover, patients with a history of PCI and smoking history had poor outcomes.

## DISCUSSION

The most common indication for TPM in this study was atrioventricular block, and the procedure time is more among patients undergoing TPM placement using the trans-jugular approach compared with the subclavian and trans-femoral approaches. Temporary cardiac pacing has different indications and the venous access site used for this can be dependent on the situation and the time period for which it might be required.^5^ Hynes et al. reported in their study that the most common venous access site for the temporary cardiac pacing was antecubital vein cutdown (59.3%) which was followed by subclavian vein (17.3%)as the second most favored site and lastly it was right internal jugular vein (10.9%) which was the least favored venous access route, but despite this the right internal jugular approach had the least number of complications in their study.^9^ While in the current study, more than half of the patients had the internal jugular vein accessed for TPM insertion with higher complications. Other studies conducted also reported, the subclavian vein as an alternative to the jugular vein as an access site for TPM and although it was associated with lesser infection than the jugular vein it had higher chances of venous thrombosis and can impair the venous access site for the permanent pacemaker. Another survey of the Italian cardiologists revealed that temporary transvenous pacing was mostly performed for brady-arrhythmias related to the conduction system of the heart, and the internal jugular vein was the preferred access site for inexperienced operators with the subclavian vein as a reasonable alternative.^10^-^12^ The femoral vein in also a common venous access site for the purpose temporary trans-venous pacing, it is associated with high complication rate, which includes infection at the site of access and deep venous thrombosis, despite this femoral vein is a common access site in some centers around the worldwide.^13^,^14^

The right internal jugular vein is the most preferred venous access site as it provides a relatively straightforward access to the right ventricle and has been associated with the least complication.^15^ In contrast although our study utilized the same right internal jugular vein as the access site for temporary cardiac pacing, but it had more complications which might be because of the difference in the skills of the operators. Chun KJ et al found in their study the trans-jugular temporary pacemaker ultimately resulted in a longer duration of the temporary pacing, similarly patients suffering from sick sinus syndrome needed a longer duration of temporary cardiac pacing as compared to the other indications of temporary pacing. (P < 0.001).^16^

The femoral route of transvenous pacing limits the mobility of the patients it is preferred in patients that require a shorter time period of temporary pacing, e.g., in Brady-arrhythmias in patients suffering from acute myocardial infarction or in patients scheduled for permanent pacemaker. In this study, the median duration of TPM was less than five days, but it was between five to 10 days in both the internal jugular and subclavian groups and more than the femoral group. While contrary to the study by Chun KJ et al,^16^ most of the patients were having acute myocardial infarctions with complete heart block who were having TPM from trans-jugular approach.

In that study, bleeding complications due to accidental arterial puncture and hemopericardium were relatively higher in the internal jugular site compared to subclavian and femoral access sites while in contrast to the current study; Chun et al reported that bleeding was more common in the femoral site. The reason for this difference could be a lack of expertise in doing trans-jugular TPM as it was not so commonly used previously in a local setup where the current study was conducted. The other common complication was the need for lead repositioning, which was needed more in the internal jugular group than the subclavian and femoral approach.

The reason for this may be the prolonged duration of temporary pacing in the internal jugular group compared to other groups results were similar to that of the study by Chun et al.^16^ A study by Irfan et al^17^ reported 34% of patients had coronary artery disease as the major cause of complete heart block. While in our study, it was 42%. Similarly, in our study, the most common indication for TPM was complete heart block 50%, which was slightly more than their findings. While in another local study performed at Rawalpindi institute of cardiology^18^ the major etiological factor of complete heart block was degenerative nodal disease rather than coronary artery disease 64% contrary to the current study.

### Limitations:

The study was a non-randomized single -center study with a relatively small sample size so the results cannot be generalized.

## CONCLUSION

Although there are different venous access sites used to insert TPMs, the most common access site was the jugular venous site. The duration of the procedure was shorter in patients with internal jugular access compared with the other sites. The trans jugular approach should be preferred in patients that require temporary pacing for more than three days.

### Authors Contribution:

**MA:** Conceived, designed and prepared the manuscript.

**SBK:** Did statistical analysis, reviewed and finalized manuscript.

**MSK:** Collected data and edited manuscript.

**ZH:** Did literature search, data collection, and data management.

**MA** and **SBK:** Take the responsibility and are accountable for all aspects of the work in ensuring that questions related to the accuracy and integrity of any part of the work are appropriately investigated and resolved.
